# XPF protein levels determine sensitivity of malignant melanoma cells to oxaliplatin chemotherapy: Suitability as a biomarker for patient selection

**DOI:** 10.1002/ijc.28454

**Published:** 2013-11-14

**Authors:** Stephanie B Hatch, Lonnie P Swift, Simona Caporali, Rebecca Carter, Esme J Hill, Thomas P MacGregor, Stefania D’Atri, Mark R Middleton, Peter J McHugh, Ricky A Sharma

**Affiliations:** 1 Department of Oncology, Oxford NIHR Biomedical Research Centre, Cancer Research UK-Medical Research Council Gray Institute for Radiation Oncology & Biology, University of OxfordOxford, Oxfordshire, OX3 7DQ, United Kingdom; 2 Department of Oncology, Weatherall Institute of Molecular Medicine, University of Oxford, John Radcliffe HospitalOxford, OX3 9DS, United Kingdom; 3 Istituto Dermopatico dell’Immacolata-IRCCS, Laboratory of Molecular Oncology, Via dei Monti di Creta 10400167, Rome, Italy

**Keywords:** DNA repair, cytotoxic chemotherapy, tissue biomarker, cell cycle

## Abstract

As the options for systemic treatment of malignant melanoma (MM) increase, the need to develop biomarkers to identify patients who might benefit from cytotoxic chemotherapy becomes more apparent. In preclinical models, oxaliplatin has activity in cisplatin-resistant cells. In this study, we have shown that oxaliplatin forms interstrand crosslinks (ICLs) in cellular DNA and that loss of the heterodimeric structure-specific endonuclease XPF-ERCC1 causes hypersensitivity to oxaliplatin in mammalian cells. XPF deficiency resulted in late S-phase arrest and persistence of double-strand breaks following oxaliplatin treatment. In a panel of 12 MM cell lines, oxaliplatin sensitivity correlated with XPF and ERCC1 protein levels. The knockdown of ERCC1 and XPF protein levels by RNA interference increased sensitivity of cancer cells to oxaliplatin; overexpression of exogenous ERCC1 significantly decreased drug sensitivity. Following immunohistochemical optimization, XPF protein levels were quantified in MM tissue samples from 183 patients, showing variation in expression and no correlation with prognosis. In 57 patients with MM treated with cisplatin or carboplatin, XPF protein levels did not predict the likelihood of clinical response. We propose that oxaliplatin should not be discarded as a potential treatment for MM on the basis of the limited activity of cisplatin in unselected patients. Moreover, we show that XPF-ERCC1 protein levels are a key determinant of the sensitivity of melanoma cells to oxaliplatin *in vitro*. Immunohistochemical detection of XPF appears suitable for development as a tissue biomarker for potentially selecting patients for oxaliplatin treatment in a prospective clinical trial.

In the United Kingdom, malignant melanoma (MM) is the fifth most common cancer and mortality rates have increased overall since the early 1970s, with increasing incidence masking improvements in diagnosis and treatment.^1^ This is representative of the global trend in fair-skinned populations around the world, in which the incidence of cutaneous MM has been increasing at a steady rate for decades.^2^ Although melanoma that has spread to distant sites is rarely curable, both ipilimumab and vemurafenib have demonstrated an improvement in progression-free (PFS) and overall survival (OS) in international, multicenter, randomized trials in patients with unresectable or advanced disease.^3^,^4^ Ipilimumab is a recombinant human antibody against the cytotoxic T-lymphocyte antigen (anti-CTLA-4), which is effective in only a minority of patients. Vemurafenib is a selective BRAF V600E kinase inhibitor, and its indication is limited to the 50% patients with unresectable or metastatic melanoma with a demonstrated *BRAF V600E* mutation. Resistance to kinase inhibitors develops within months^5^; although combination of these agents,^6^ or combination with cytotoxic chemotherapy^7^ may extend overall survival.

It is therefore clear that cytotoxic chemotherapy remains an important option for the treatment for patients with MM. The objective response rate to dacarbazine (DTIC) and the nitrosoureas, carmustine (BCNU) and lomustine, is ≈10–20%.^8^ Other agents with modest single-agent activity include vinca alkaloids, cisplatin, and taxanes.^9^ Tests for selecting patients for particular cytotoxic chemotherapies do not currently exist, but the successful development of validation of such tests could significantly improve objective response rates for patients with MM.

**What’s new?**

With options for systemic treatment of malignant melanoma (MM) on the rise, there is an increasing need to develop biomarkers for patient selection. To that end, this study explored the possibility of a biomarker to improve objective response rates to the drug oxaliplatin. The study reveals a mechanism by which mammalian cells are rendered hypersensitive to oxaliplatin that centers around the loss of endonuclease XPF-ERCC1. Sensitivity to oxaliplatin was directly related to XPF and ERCC1 protein levels. The findings indicate that XPF may be a suitable biomarker for MM patient selection for oxaliplatin therapy.

Elevated expression of DNA repair genes has been reported in primary melanomas that subsequently went on to metastasize when compared with nonrecurrent primary tumors.^10^ This increased expression could contribute to clinical resistance shown by melanoma to conventional DNA-damaging chemotherapeutics such as dacarbazine and cisplatin. Consistent with this hypothesis, levels of certain DNA repair proteins may be prognostic biomarkers in patients with MM, *e.g*. XRCC5 (Ku80), required for DNA double-strand break repair, has been associated with significantly worse survival.^11^

Although the response rate of colorectal cancer to cisplatin chemotherapy is too low to justify its widespread clinical use, oxaliplatin (1R, 2R-diaminocyclohexane oxalatoplatinum(II)) demonstrated significant activity against MM and colorectal cancer cell lines in anticancer drug screening carried out by the US National Cancer Institute, including cell lines resistant to cisplatin.^12^–^14^ These findings led clinical investigators to perform a clinical trial of the combination of oxaliplatin, docetaxel and sargramostim (GM-CSF) in patients with previously treated MM.^15^ This small clinical study demonstrated limited clinical activity, but the investigators did not consider any means of patient selection to improve objective response rates. A further study of oxaliplatin, bevacizumab and sorafenib in patients with MM has not yet been reported in full.^16^

Based on preclinical reports of the activity of oxaliplatin in treating MM, we wished to study the mechanism of action of the drug in MM cells and in cells with specific DNA repair defects. Our results suggest that oxaliplatin forms biologically relevant interstrand crosslinks (ICLs) in cellular DNA and that loss of the heterodimeric structure-specific endonuclease, XPF-ERCC1, determines the sensitivity of MM cells to oxaliplatin. Furthermore, we develop XPF staining by immunohistochemistry (IHC) as a clinical test to be validated for patient selection in clinical trials of oxaliplatin treatment in patients with MM.

## Material and Methods

*In vitro* detection of drug-DNA crosslinks was performed as previously described.^17^ The plasmid pYes 2.0 (Life Technologies) was linearized with NotI and 3′-end-labeled with [α-32P]dGTP or [α-32P]dCTP in the presence of the Klenow fragment of *Escherichia coli* DNA polymerase I. Unincorporated label was removed using G-50 ProbeQuant columns and the labeled DNA was re-suspended in salmon sperm DNA in TE buffer. End-labeled DNA (25 µM bp) was reacted with drugs at 37°C for 1 hr in PBS. Unreacted drugs were extracted with phenol and chloroform, and the DNA was precipitated in ethanol, heat denatured (to separate non-crosslinked DNA from crosslinked DNA) then separated in an 0.8% agarose gel (1× TAE buffer [40 mM Tris acetate, 1 mM EDTA]) at 45 V for 16 hr. Gels were analyzed on a Typhoon imaging instrument (GE Healthcare). The Comet assay was performed as previously described.^18^ Samples were divided into two with one half being irradiated with 10 Gy X-irradiation. Samples were mixed with low melting temperature agarose and set on microscope slides, lysed, washed and subjected to electrophoresis in alkali conditions. DNA was stained and the tail moment was measured for 50 comets per slide using Komet Assay Software (Kinetic Imaging, Liverpool, UK).

The cell lines used in this study were primary cell culture derived from patients.^19^,^20^ Protein extracts from 12 MM cell lines were probed for XPF and ERCC1 levels by Western blotting. Whole-cell lysates were collected in modified RIPA lysis buffer [50 mM Tris pH 7.4, 150 mM NaCl, 1% NP-40, 1 mM EDTA, 0.1% SDS plus 1× protease inhibitor cocktail (Roche), 1 mM DTT, 1:100 phosphatase inhibitor cocktail 2 (Sigma Aldrich)] and equal amounts of total protein were loaded and run under standard conditions.

Approval for this project was obtained from Oxfordshire Research Ethics Committee C for research involving human tissue. Immunohistochemical staining using the Leica Bond-Max machine at 1:200 dilution with antigen retrieval using the standard pH 9.0 buffer for 10 min. XPF nuclear staining was scored according to the intensity of the staining (0 = no staining, 1 = weak staining, 2 = moderate staining, 3 = strong staining) and the percentage of nuclei staining (0 = 0%, 1 = <10%, 2 = 10–50%, 3 = 50–80%, 4 = >80%) by two independent investigators who were blinded to the clinical and pathological characteristics of the patients. A consensus score was agreed for intensity score and percentage score for each core and a composite score was derived (ranging from 0 to 12) using the product of the two scores. Tissue microarrays were prepared in Oxford, UK (183 patients) and the Karolinska Institute, Stockholm, Sweden (57 patients). The median age of patients at the time of MM diagnosis was 58 years, 139 of whom had cutaneous melanoma, the remainder having other primary sites (head and neck 33, acral 7, mucosal 2, unknown 2). For the 183-patient cohort, the majority of patients had nonmetastatic disease at the time of diagnosis (Stage 1 = 29 patients, Stage 2 = 81 patients, Stage 3 = 56 patients, Stage 4 = 8 patients, not known = 9 patients). None of the patients in this cohort received cisplatin, carboplatin or oxaliplatin chemotherapy. A cutoff for median composite Score of 6 was used to subclassify the cores as having low XPF expression (0–6) or high XPF expression.^7^–^12^ Statistical analysis was performed using SPSS version 20 (specific tests stated in the figure legends).

## Results and Discussion

### Oxaliplatin forms ICLs in mammalian cells

**Figure 1:**
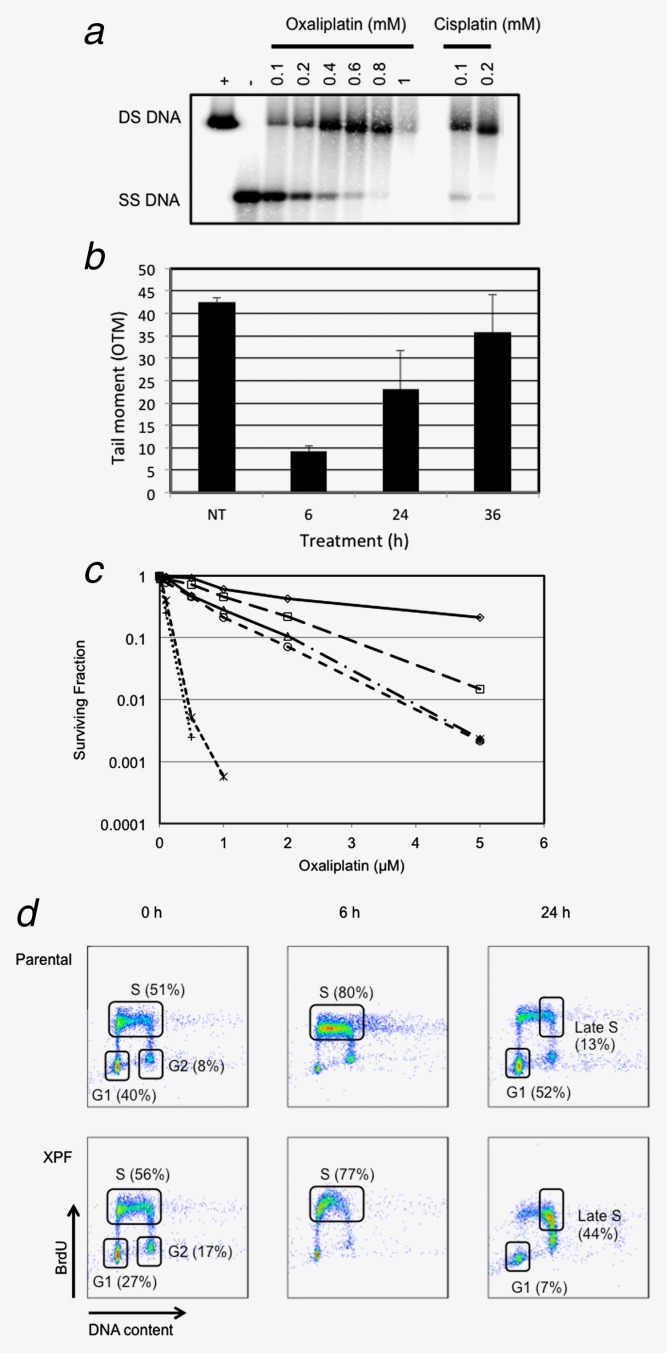
Oxaliplatin forms ICLs in DNA *in vitro*; cells deficient in XPF or ERCC1 are hypersensitive to the drug and arrest in the S-phase of the cell cycle. (*a*) *In vitro* detection of drug-DNA crosslinks. The migration of DNA as single-stranded DNA (SS DNA) indicates no DNA crosslinks formed compared to crosslinked DNA that migrates as double-stranded DNA (DS DNA). (*b*) Modified Comet assay to measure formation of drug-DNA crosslinks. The parental Chinese Hamster Ovary cell line AA8 was treated with 250 µM oxaliplatin for 4 hr and then treated with ionizing radiation (IR), as previously described.^18^ The initial Olive Comet tail moment (OTM) observed in cells not treated (NT) with oxaliplatin indicates IR-induced DNA breaks. Following oxaliplatin treatment, drug-DNA crosslinks are formed which retard the IR-induced Comet tail. The Comet tail returns to control (NT) levels over this time period consistent with processing and ICL unhooking.^18^ Bars represent three independent experiments and error bars show SD. (*c*) Chinese Hamster Ovary repair mutant cell lines AA8 (WT 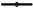), UV23 (XPB 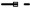), UV135 (XPG 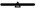), UV40 (FancG 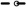), UV96 (ERCC1 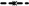) and UV47 (XPF 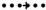) were seeded at a density of 1000 cells per 100 mm Petri dish in 15 ml of complete media. Cells were allowed to attach overnight before being treated with various concentrations of oxaliplatin and incubated for 8 days to allow colonies to form. Cells were fixed and stained as previously described.^21^ Colonies (being greater than 50 cells) were counted on an Oxford Optronic Col Count Instrument. (*d*) CHO parental (AA8) and XPF repair mutant (UV47) cells were treated with 10 μM oxaliplatin for 2 hr and then analyzed, at the indicated times after treatment, for cell cycle distribution using the BrdU incorporation method and reagents as previously described.^21^ The *y*-axis indicates incorporation of BrdU as a measure of active synthesis where the *x*-axis is a measure of DNA content. G1, S and G2-phase populations are highlighted in the parental 0 hr plot where the S-phase population is defined as an early through to a late being the population shift from the left through to the right of the designated region.

Using an *in vitro* assay, the extent of ICL formation by oxaliplatin and cisplatin crosslinks was determined (Fig. 1*a*). Oxaliplatin was less efficient than cisplatin at crosslinking DNA, requiring approximately six times the concentration of drug to form the same level of crosslinks (Fig. 1*a*). These results are consistent with a study in H460 tumor cells in which cisplatin treatment produced more crosslinks in DNA than equimolar concentrations of oxaliplatin, measured by modification of the alkaline Comet assay.^22^ Oxaliplatin inhibits DNA synthesis more than cisplatin, and kills a greater number of cells per crosslink formed.^23^,^24^

Using the modified Comet assay, we treated Chinese Hamster ovary (CHO) cells with oxaliplatin and measured ICL formation and repair following ionizing radiation (IR)-induced DNA breaks (Fig. 1*b*). Following oxaliplatin treatment, drug-DNA crosslinks are formed which retard the IR-induced Comet tail. In our experiments, the Comet tail returned to control levels over 36 hr, a timescale consistent with ICL processing and unhooking.^18^

### XPF deficiency is associated with hypersensitivity to oxaliplatin and S-phase arrest

To identify key DNA repair proteins that cause hypersensitivity to oxaliplatin chemotherapy, we screened CHO cell lines with mutations in the NER proteins XPB, XPG, XPF, ERCC1 and FancG by clonogenic assay. As shown in Figure 1*c*, XPF and ERCC1 mutant cells were ≈30 times and 15 times, respectively, more sensitive to oxaliplatin than parental cells, with IC_50_ values of ≈40 nM for XPF mutant cells and 80 nM for ERCC1 mutant cells.

The pattern of relative sensitivity observed for mutant XPF and ERCC1 CHO cell lines observed in Figure 1*c* was consistent with that observed using crosslinking agents such as nitrogen mustard (HN2) and mitomycin C (MMC), which form ICLs in DNA.^18^,^25^,^26^ ICLs effectively block replication and transcription and their repair requires proteins from multiple DNA repair pathways, including NER, BER, double-strand break (dsb) repair and the Fanconi Anemia repair pathway.^27^ Processing of ICLs requires initial nicking incisions to release the crosslinked nucleotides on one strand in a process termed “unhooking.” The current consensus is that unhooking involves the XPF-ERCC1 heterodimeric structure-specific endonuclease.^28^

On the basis of these observations, we sought to find further biological evidence that ICLs were formed in cells following treatment with oxaliplatin. We treated CHO cells with oxaliplatin for 2 hr and subsequently studied cell cycle progression by flow cytometry using the bromo-deoxyuridine (BrdU) method. Twenty-four hours after treatment, whereas wild-type cells re-entered the normal cell cycle, as shown in Figure 1*d*, cells deficient in XPF demonstrated persistent late S-phase arrest. This finding was consistent with the hypothesis that the cells deficient in XPF had difficulty coping with lesions created in DNA by oxaliplatin, such as ICLs.

### XPF deficiency results in persistence of double-strand breaks following oxaliplatin treatment

**Figure 2:**
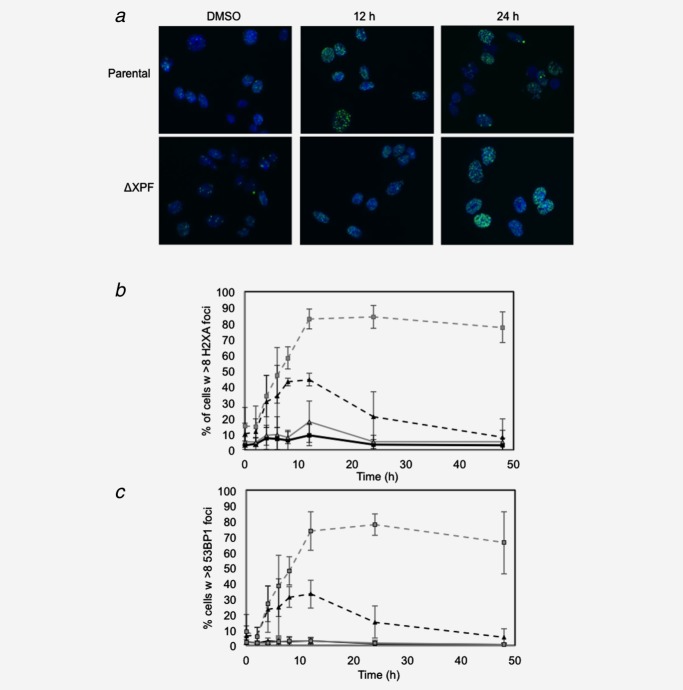
In the absence of XPF, oxaliplatin treatment causes persistence of double-strand breaks in mammalian cells. (*a*) CHO cells (10,000) were treated with 10 µM oxaliplatin (OX) for 2 hr in a 96-well plate and fixed in 4% paraformaldehyde at various time points, blocked with 0.1% Triton X-100 and 1% bovine serum albumin (BSA) in PBS before being stained for γH2AX (*b*) and 53BP1 (*c*) foci (1:2000 for γH2AX and 1:1000 for 53BP1 in 1% BSA) overnight. A 1:500 dilution of Alexa fluor 488-labeled secondary antibody was diluted in 1% BSA in PBS and incubated for 1 hr at room temperature, and then cells were stained with a 1:2000 dilution of 1 mg/ml DAPI (Sigma Aldrich) for 10 min at RT before being analyzed on IN Cell Analyser 1000 (GE Life Sciences), counting cells with more than 8 foci per nucleus. Data (*b*,*c*) are plotted versus time for WT DMSO (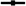), WT 10 µM oxaliplatin (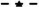), XPF DMSO (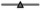) and XPF 10 µM oxaliplatin (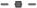). (*n* = 3, error bars = standard deviation).

During replication-coupled ICL repair, DNA dsbs form that are ultimately repaired by translesion synthesis and homologous recombination (HR). Previous studies have suggested that rodent and human cells lacking ERCC1-XPF accumulate replication-associated dsbs when treated with MMC or HN2.^18^,^27^,^29^ We therefore studied the kinetics of dsb formation and repair following oxaliplatin treatment of cells deficient in XPF. CHO cells proficient and deficient in XPF were treated with oxaliplatin and nuclear foci of γH2AX and 53BP1 proteins were quantified (Figs. 2*a*–2*c*). Both γH2AX and 53BP1 associate early with break-ends during the dsb response and are well-established markers of dsb induction. Consistent with the hypothesis that oxaliplatin induces ICLs that have biological consequences for mammalian cells, XPF-deficient cells had 9.7-fold (γH2AX, *p* ≤ 0.001) or 12.9-fold (53BP1, *p* ≤ 0.01) higher levels of dsbs 48 hr after oxaliplatin treatment (Figs. 1*b* and 1*c*) measured by both indices, with *p* values determined by two-tailed, paired *t*-test. Collectively, these data clearly demonstrate for the first time in mammalian cells that oxaliplatin treatment results in the creation of dsbs and that deficiency of XPF results in significant persistence of unrepaired dsbs.

### XPF protein levels correlate with sensitivity of MM cells to oxaliplatin

**Figure 3:**
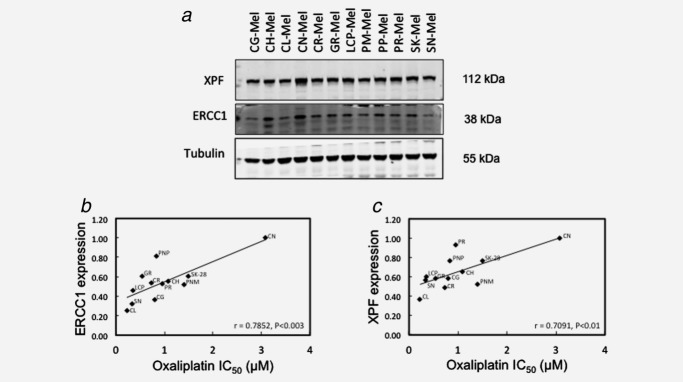
Significant correlation between protein levels of XPF or ERCC1 and oxaliplatin sensitivity in human MM cells. (*a*) Protein extracts from 12 MM cell lines were probed for XPF and ERCC1 levels by Western blotting. Oxaliplatin IC_50_ values were determined by clonogenic assays. Briefly, melanoma cells were exposed to graded concentrations of oxaliplatin for 24 hr. After 10–11 days of incubation in the absence of the drug, colonies were fixed and stained with crystal violet for visualization. Only those colonies containing 50 or more cells were scored as survival colonies. IC_50_ values were calculated on the regression line in which colony formation efficiency was plotted against the logarithm of drug concentration. Protein expression levels were quantified by Odyssey image analysis (Li-cor Biosciences, Cambridge, UK) for ERCC1 (*b*) and XPF (*c*), corrected for tubulin expression and plotted against oxaliplatin IC_50_ values determined in the cell lines and the correlation measured (*n* = 3). Oxaliplatin IC_50_ values were determined by clonogenic assays.

If XPF-ERCC1 protein levels are a key determinant of oxaliplatin sensitivity in human cancer cells, one might expect oxaliplatin sensitivity to correlate with constitutive levels of these repair proteins. We measured XPF and ERCC1 protein levels in whole cell lysates from 12 cell lines derived from patients with MM and studied correlations with oxaliplatin IC_50_ values measured by clonogenic assay. XPF protein levels varied approximately threefold across the cell lines (Fig. 3*a*) and, as one would expect for binding partners, there was a strong correlation between both XPF and ERCC1 protein expression across the 12 cell lines (*r* = 0.749, *p* < 0.005). Importantly, as shown in Figure 3*b*, a statistically significant correlation was observed between oxaliplatin IC_50_ values and protein expression of either XPF (*r* = 0.71, *p* < 0.01) or ERCC1 (*r* = 0.79, *p* < 0.003). Using exactly the same experimental design to treat the cell lines with cisplatin demonstrated that this significant correlation only applied to oxaliplatin, not to cisplatin (Supporting Information Figure 1).

**Figure 4:**
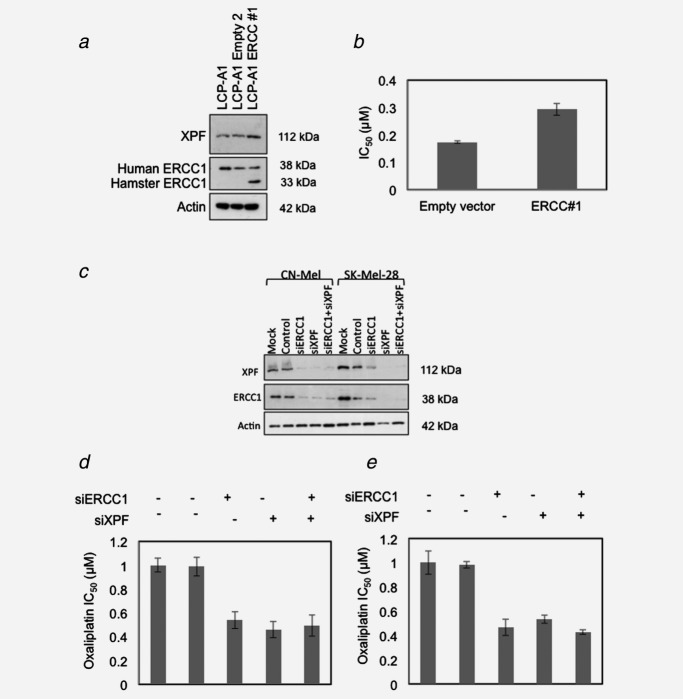
Attenuation of ERCC1 or XPF levels in cells alters sensitivity to oxaliplatin treatment. (*a*) The plasmid pEf6-V5-His-ERCC1, carrying the hamster ERCC1 cDNA, and the pEF6-V5-His-Topo empty vector^30^ were stably transfected into the LCP-Mel-A1 cell clone, obtained from the melanoma cell line LCP-Mel by limiting dilution. Blasticidin (Invitrogen) was used at 3 mg/ml to maintain expression of the vectors. To assess the expression of ERCC1 and XPS in the transfected clones, Co-IP of whole-cell lysates were collected (50 mM Tris pH 7.4, 150 mM NaCl, 1% NP-40, 0.1% SDS) and then cleared by centrifugation before incubation with antibodies (anti-XPF Abcam SPM228, Anti-hERCC1 Santa Cruz sc-17809. (*b*) Clonogenic assays (as described above) were used to calculate the IC_50_ (*n* = 3, error = standard deviation). (*c*–*e*) CN-Mel and SK-Mel-28 cells were mock transfected or transfected with 10 nM siERCC1 (CCCGGGTGACTGAATGTCTGA), siXPF (CTCCTTGATGCACCACGTTAA) or both or control siRNA (AllStars Negative Control siRNA) (all purchased from Qiagen) using Lipofectamine™ RNAiMAX Reagent (Invitrogen Corporation). After 72 hr of culture at 37°C, the cells were recovered for Western blot analysis of XPF and ERCC1 expression (*c*) and for evaluation of oxaliplatin sensitivity by clonogenic assays, as described above (*d*, CN-Mel; E, SK-Mel-28).

We then transfected a low-expressing MM cell line with a vector containing ERCC1. This experiment demonstrated that ectopic expression of ERCC1 into a human MM cell line could also stabilize XPF (Fig. 4*a*) and that it significantly increased the oxaliplatin IC_50_ (*p* ≤ 0.01 by two-tailed, paired *t*-test) as shown in Figure 4*b*. Furthermore, we depleted XPF and ERCC1 protein levels by RNA interference in two MM cell lines, CN-Mel and SK-Mel-28 (Figs. 4*c*–4*e*). siRNA-mediated depletion of ERCC1 and XPF increased sensitivity to oxaliplatin by an average of 52% (*p* ≤ 0.01) for SK-Mel-28, and by 50% (*p* ≤ 0.05) for CN-Mel.

These data demonstrate for the first time that modulation of XPF and ERCC1 protein levels modifies the oxaliplatin sensitivity of human melanoma cells. The results are consistent with treatment of non-small cell lung cancer, ovarian and breast cancer cells with cisplatin.^31^ The use of clonogenic survival endpoints in this study appear to be more sensitive than previous studies using proliferation assays to assess oxaliplatin treatment.^32^

### Measurement of XPF protein levels in human melanoma tissue as a potential biomarker for patient selection

A current priority in translational cancer research is the development of clinical biomarkers to select patients for cytotoxic and biological anticancer therapies. It is important that candidate biomarkers are key determinants of sensitivity to the agent being studied and that the biological relationship between the biomarker and the therapeutic agent is understood. Since XPF protein levels fulfill these criteria for oxaliplatin chemotherapy, we studied the specificity, reproducibility and variability of XPF protein expression by IHC of tissue microarray samples from 183 patients with MM.

**Figure 5:**
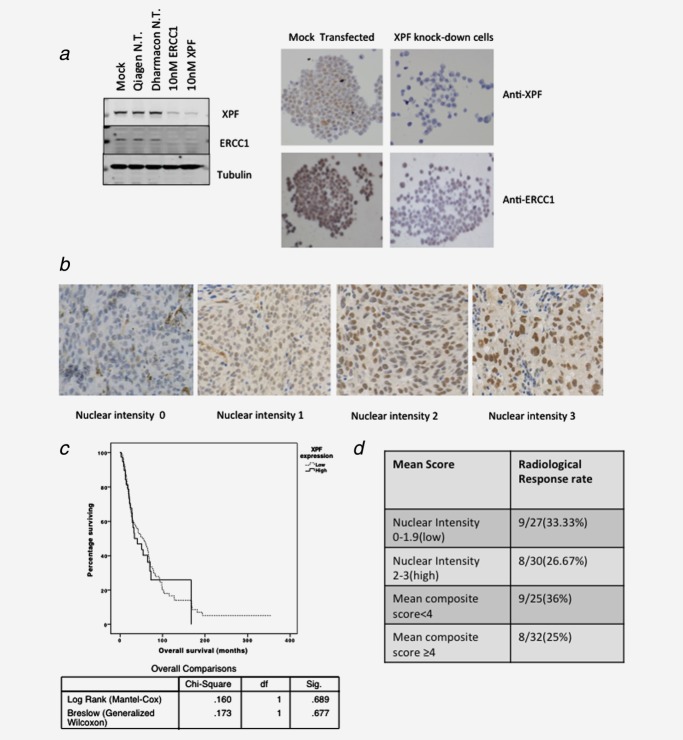
Evaluation of XPF protein as a potential biomarker for sensitivity to platinum chemotherapy in paraffin-embedded human melanoma tissue. (*a*) Validation of anti-XPF and anti-ERCC1 antibodies for IHC on paraffin-embedded ST16 gastric carcinoma cells. Western blot showing effect of XPF and ERCC1 protein expression in ST16 cell lysates after XPF and ERCC1 knockdown using the Lipofectamine RNAiMax Reagent (Invitrogen, Carlsbad, CA; 13778-150), performed according to the manufacturer’s protocol using 10 nM of siRNA(Qiagen N.T. is nontargeting SiRNA). ST16 cell pellets were stained with antibodies against XPF (clone SPM228, Abcam, Cambridge, MA) with antigen retrieval in a water bath at 99°C, using 1 mM ethylene diaminetetraacetic acid (EDTA) buffer (pH 8.0). Primary antibody was diluted 1:100 and incubated for 2 hr at room temperature. A horseradish peroxidase based system (EnVision+; Dako, Glostrup, Denmark) was used for detection of primary antibody binding. RNAi knockdown of XPF in ST16 cells produced decreased staining for XPF when compared to a mock transfection. (*b*) Staining for XPF on a melanoma tissue microarray, showing a variation in the levels of these proteins. (*c*) Kaplan-Meier analysis of the effect of XPF expression on overall survival in 183 patients with melanoma. (*d*) Analysis for an association between mean XPF nuclear intensity score/mean composite score and radiological response in tissue microarrays, representing cores from 57 patients with metastatic melanoma treated with cisplatin/carboplatin chemotherapy. Primary tissues were obtained from patients with MM prior to cisplatin or carboplatin chemotherapy (cutaneous = 45 patients, ocular = 3 patients, mucosal = 2 patients, unknown primary location = 7 patients). The median nuclear intensity score (2) and median composite score (4) were used to subclassify the cores into two groups. Analysis for an association between RECIST response^33^ and XPF score were performed using the Pearson’s chi-square test and odds ratios were calculated in SPSS version 20. Neither nuclear intensity score (*α*² = 0.302, *p* = 0.583, OR = 1.375; 95% CI = 0.441–4.291) nor composite XPF score (*α*² = 0.811, *p* = 0.368, OR = 1.688; 95% CI = 0.538–5.294) were associated with clinical response.

We first demonstrated the specificity of the anti-XPF antibody and reproducibility of IHC staining using an automated staining system (Figs. 5*a* and 5*b*). Our experiments with all commercially available anti-ERCC1 antibodies demonstrated excessive nonspecific staining of human MM tissue, so we did not proceed with the development of ERCC1 as an IHC biomarker. Having optimized anti-XPF staining, we evaluated XPF protein expression in 213 cores of MM tissue on microarrays representing 183 patients with MM not treated with platinum chemotherapy, comprising tissue from resected primary melanomas and resected lymph node metastases. On analysis of these samples, XPF protein expression was not prognostic (Fig. 5*c*). Other clinical markers, however, that were not related to XPF protein levels, were prognostic such as thickness of the primary tumor, presence of ulceration and disease stage (HR = 1.12, 95% CI = 1.01–1.25, *p* = 0.03 for tumor thickness; HR = 2.24, 95% CI = 1.34–3.74, *p* = 0.002 for ulceration; HR = 4.91 for Stage III/IV, 95% CI = 2.17–11.12, *p* < 0.001).

Finally, we wished to explore whether XPF expression might have potential value as a predictive biomarker for sensitivity to platinum therapy in patients with MM. According to our enquiries, tissue samples from melanoma patients treated with oxaliplatin have never been biobanked anywhere in the world. We therefore studied XPF protein expression by IHC of tissue microarray samples from 57 patients with MM treated with cisplatin or carboplatin containing chemotherapy for whom radiological response data from computed tomography scans were available. None of these patients received oxaliplatin chemotherapy. Two tissue cores were available for the majority of these patients and we derived mean nuclear intensity and mean composite score values for each patient to analyze the scores for a potential association with radiological response (Fig. 5*d*). Analysis of these clinical specimens demonstrated that XPF protein expression was not predictive for clinical response to cisplatin/carboplatin chemotherapy.

The staining and scoring system developed in this part of the project demonstrates the potential utility of XPF as a predictive biomarker for oxaliplatin sensitivity in patients with MM. We advocate extension of this study to a larger cohort of samples with a view to validation of XPF as a selection biomarker in clinical trials of oxaliplatin chemotherapy in patients with MM. This would involve further validation of XPF in an additional, large, independent cohort of patients with MM, a prospective study with oxaliplatin-based chemotherapy in unselected patients in which XPF expression could be performed in real time and correlated with response, and finally a prospective study in which patients would be selected for oxaliplatin chemotherapy versus alternative therapy based on XPF expression.

In summary, our data suggest that oxaliplatin should not be discarded as a potential treatment for MM on the basis of the limited activity of cisplatin in unselected patients. Our aim was to develop a biomarker to identify a subset of patients that may benefit from oxaliplatin chemotherapy. We show that XPF-ERCC1 protein levels are a key determinant of sensitivity to oxaliplatin chemotherapy and that the mechanism of cytotoxicity appears to be related to ICL repair. Immunohistochemical detection of XPF appears suitable for development as a tissue biomarker for selecting patients for oxaliplatin treatment in a clinical trial. As the options for systemic treatment of MM increase, our results, if fully validated as described above, offer a means to identify patients who might benefit from the addition of oxaliplatin to combination regimens or as an additional line of therapy.
