# Supplementary material for: XPF protein levels determine sensitivity of malignant melanoma cells to oxaliplatin chemotherapy: Suitability as a biomarker for patient selection
Source: Int J Cancer. 2013 Nov 14;134(6):1495–503. doi: 10.1002/ijc.28454 (PMC4233955; doi:10.1002/ijc.28454)
Supplement: Supplementary file 1 [file ijc0134-1495-sd1.pdf]

Supplementary Figure 1

A

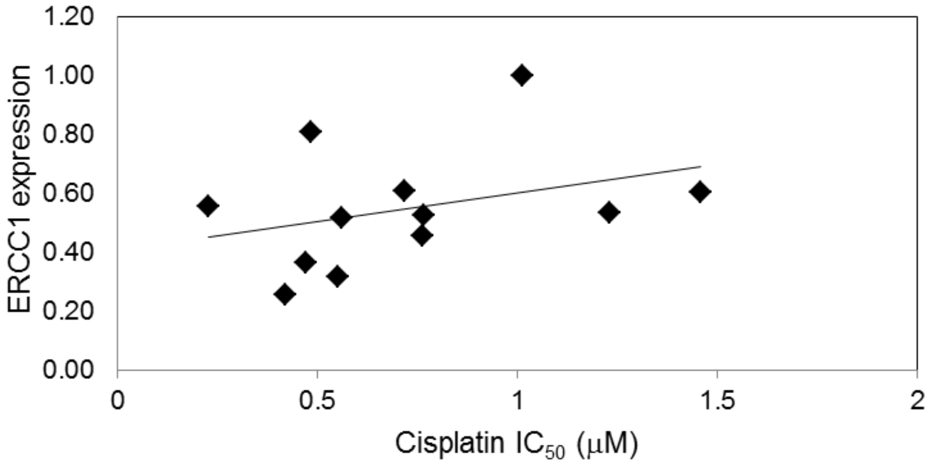

B

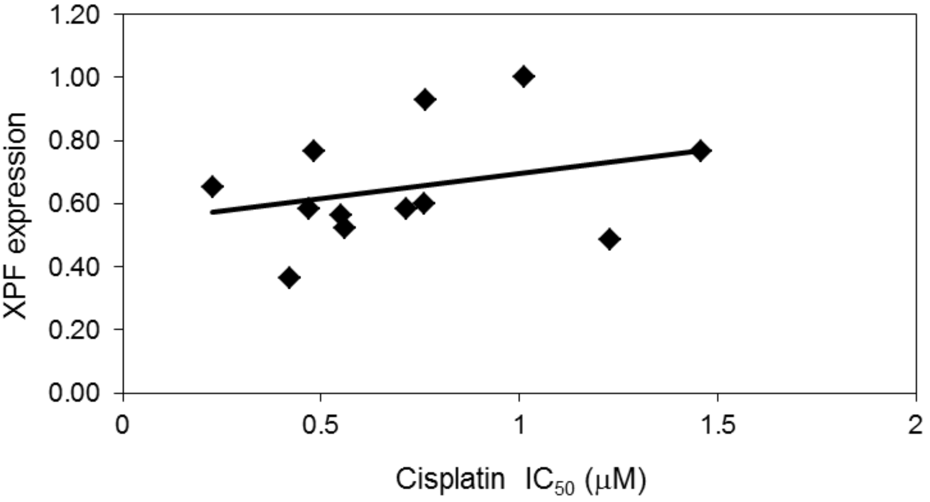

Lack of significant correlation between protein levels of XPF or ERCC1 and cisplatin sensitivity in human malignant melanoma cells. Melanoma cells were exposed to graded concentrations of cisplatin for 24 h. After 10-11 days of incubation in the absence of the drug, colonies were fixed and stained with crystal violet for visualization. Only those colonies containing 50 or more cells were scored as survival colonies. IC_{50} values were calculated on the regression line in which colony formation efficiency was plotted against the logarithm of drug concentration. Quantified protein expression levels for ERCC1 (A) and XPF (B) (shown in Figure 2A) were plotted against cisplatin IC_{50} values determined in the cell lines and the correlation measured (n = 3).
